# Transcriptome and Quasi-Targeted Metabolome Analyze Overexpression of 4-Hydroxyphenylpyruvate Dioxygenase Alleviates Fungal Toxicity of 9-Phenanthrol in *Magnaporthe oryzae*

**DOI:** 10.3390/ijms23137116

**Published:** 2022-06-27

**Authors:** Yi Wang, Ziyi Wang, Sauban Musa Jibril, Mian Wei, Xin Pu, Chao Yang, Chan Ma, Qi Wu, Lina Liu, Yiji Quan, Chengyun Li

**Affiliations:** State Key Laboratory for Conservation and Utilization of Bio-Resources in Yunnan, Yunnan Agricultural University, Kunming 650201, China; wyi_0114@ynau.edu.cn (Y.W.); wziyi0215@163.com (Z.W.); saubanzango@gmail.com (S.M.J.); wm2019310510@163.com (M.W.); puxin_1998@163.com (X.P.); yangchao78833@163.com (C.Y.); mc322074@163.com (C.M.); wuqi@ynau.edu.cn (Q.W.); handyliu@126.com (L.L.); qyi_28@163.com (Y.Q.)

**Keywords:** *Magnaporthe oryzae*, 9-phenanthrol, 4-hydroxyphenylpyruvate dioxygenase, overexpression, tyrosine degradative pathway

## Abstract

*Magnaporthe oryzae*, the causal agent of rice blast disease, produces devastating damage to global rice production. It is urgent to explore novel strategies to overcome the losses caused by this disease. 9-phenanthrol is often used as a transient receptor potential melastatin 4 (TRPM4) channel inhibitor for animals, but we found its fungal toxicity to *M. oryzae*. Thus, we explored the antimicrobial mechanism through transcriptome and metabolome analyses. Moreover, we found that overexpression of a gene encoding 4-hydroxyphenylpyruvate dioxygenase involved in the tyrosine degradative pathway enhanced the tolerance of 9-phenanthrol in *M. oryzae*. Thus, our results highlight the potential fungal toxicity mechanism of 9-phenanthrol at metabolic and transcriptomic levels and identify a gene involving 9-phenanthrol alleviation. Importantly, our results demonstrate the novel mechanism of 9-phenanthrol on fungal toxicity that will provide new insights of 9-phenanthrol for application on other organisms.

## 1. Introduction

Rice (*Oryza sativa*) is an important crop widely grown in the world. However, rice production is seriously endangered by a variety of pathogens throughout the growing season, which threatens food security [1]. Rice blast disease, caused by ascomycetes fungus *Magnaporthe oryzae*, is a major constraint to rice production. *M. oryzae* has been listed as the top plant pathogen, and the wheat infecting pathotype causes the yield losses of global wheat production [2,3]. Therefore, developing new strategies for blast disease management is necessary.

9-phenanthrol, also named 9-hydroxyphenanthrene, phenanthrene-9-ol, or 9-phenanthrenol (C_14_H_10_O), is an aromatic compound from phenanthrene. 9-phenanthrol is a widely used TRPM4 (transient receptor potential melastatin 4) channel inhibitor for animals [4]. TRPM4 channel is a calcium-activated, phosphatidylinositol-4,5-bisphosphate (PtdIns(4,5)P_2_)-modulated, non-selective cation channel that belongs to the family of melastatin-related transient receptor potential (TRPM) channels. TRPM4 is involved in important physiological processes such as Ca^2+^-dependent immune response and human heart conduction dysfunction [5]. 9-phenanthrol is the degradation intermediate of phenanthrene, suggesting its risk of environmental toxicity. Previous results indicated that 9-phenanthrol has high sorption and heterogenous properties with lower risks for 9-phenanthrol to phenanthrene [6]. However, the antifungal activity of this compound was hardly reported. A paper reported that 9-phenanthrol could change the colony color of *Stemphylium sarcinaeforme* and inhibit the spores of *Monilinia fructicola* in the 1950s [7]. There were no other reports of 9-phenanthrol involving antifungal mechanisms, implying there are many novel characters about 9-phenanthrol waiting to be revealed.

In this paper, we tested the fungal toxicity of 9-phenanthrol on *M. oryzae* and rice blast disease management through transcriptome and metabolome and found that a gene encoding 4-hydroxyphenylpyruvate dioxygenase could play an important role in the tolerance of 9-phenanthrol in *M. oryzae*. Our results provide the antifungal mechanism of 9-phenanthrol, which could be considered a novel fungicide for disease management.

## 2. Results

### 2.1. 9-Phenanthrol Displayed Antifungal Activity against M. oryzae

**Figure 1:**
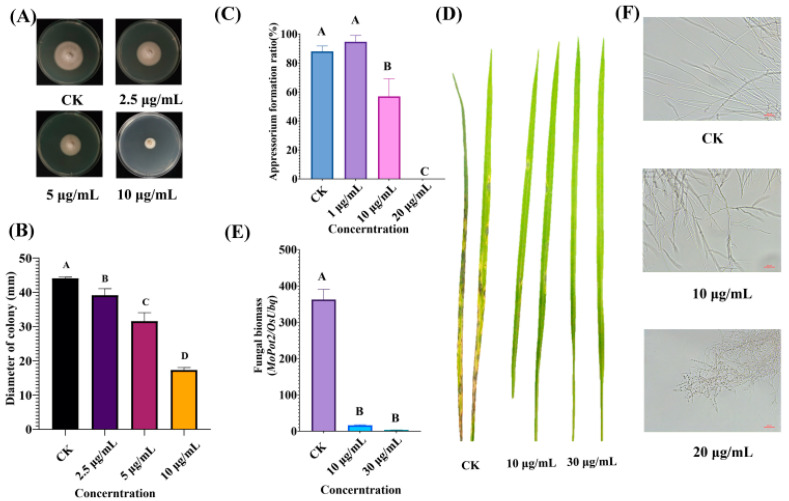
The inhibitory effects of 9-phenanthrol on blast growth and pathogenicity. (**A**) The effect of 9-phenanthrol on mycelial growth (**A**,**B**) and appressorial formation (**C**). Application of 9-phenanthrol decreases the pathogenicity and biomass of *M. oryzae* (**D**,**E**). The morphological changes of hypha with 9-phenanthrol treatment (**F**). Values are presented as the mean of results of triplicate experiments ± SD. Bars with different capital letters indicate significant differences between treatments at a *p*-value < 0.01 using a Duncan statistics method.

9-phenanthrol was reported to have toxicity to the spores of *M. fructicola*; thus, we tested its antifungal activity against *M. oryzae*. The results showed that 9-phenanthrol application could significantly inhibit the mycelial growth (Figure 1A,B). The formation of appressorium is a key step in deciding the infection of *M. oryzae*. We found that 9-phenanthrol disrupted the appressorium formation at 10 μg/mL (Figure 1C). Moreover, the spore suspensions mixed with 9-phenanthrol were used to inoculate rice seedlings, and the results showed that the number of lesions was much less than those without 9-phenanthrol treatment (Figure 1D). We calculated the fungal biomass of rice seedlings with different treatments, and similar results showed that the fungal biomass was significantly decreased treating with 10 μg/mL and 30 μg/mL 9-phenanthrol (Figure 1E). We observed the increased branches and septum of hypha and abnormal mycelium after 9-phenanthrol treatment (Figure 1F). Our results clearly suggested that 9-phenanthrol inhibits the fungal development and infection in *M. oryzae*.

### 2.2. Transcriptome Analysis Reveals Gene Ontology Categories and KEGG Enrichment Analysis of DEGs

**Figure 2:**
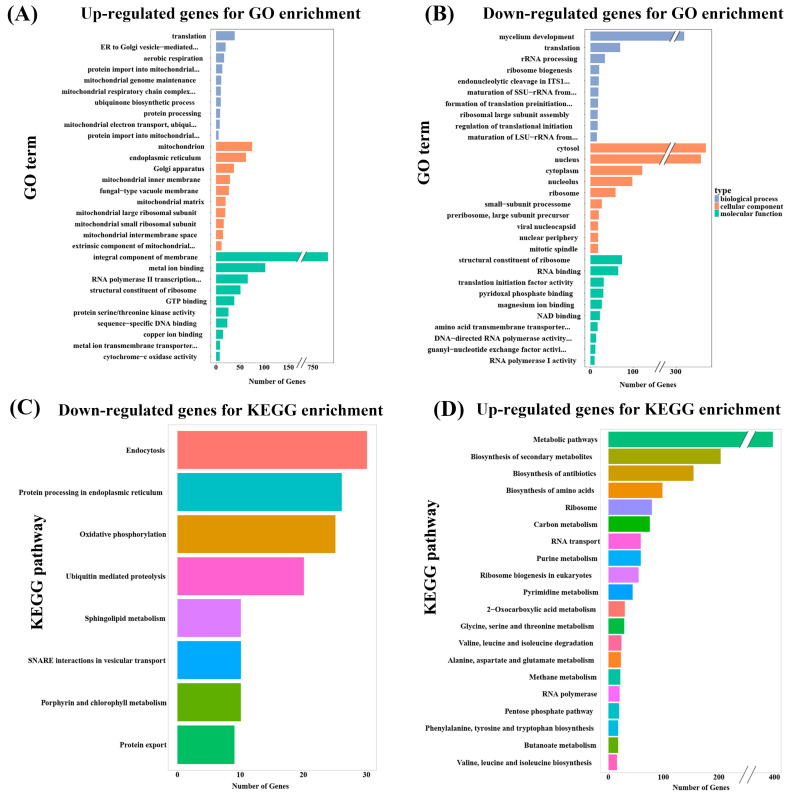
Gene Ontology (GO) and Kyoto Encyclopedia of Genes and Genomes (KEGG) enrichment analyses of differently expressed genes (DEGs) in *M. oryzae* treated with 9-phenanthrol. The GO enrichment of upregulation (**A**) and downregulation (**B**) of DEGs. The KEGG analysis of upregulation (**C**) and downregulation (**D**) of DEGs.

In order to reveal the inhibitory mechanism of 9-phenanthrol on *M. oryzae*. We conducted a high-throughput RNA sequencing to obtain transcripts from the *M. oryzae* Guy11 mycelia treated with 9-phenanthrol. Compared with control, a total of 6310 differentially expressed genes (DEGs) and 3102 upregulated and 3208 downregulated genes were identified in *M. oryzae* under 9-phenanthrol treatment (Table S1). To comprehend the function of DEGs, we performed a Gene Ontology (GO) enrichment analysis of the DEGs (Figure 2A,B, Tables S2 and S3). The upregulated genes involving biological processes were mainly associated with translation, ER (endoplasmic reticulum) to Golgi vesicle-mediated transport, aerobic respiration, and protein import into the mitochondrial inner membrane. In contrast, genes related to mycelium development, translation, ribosome biogenesis, and rRNA processing were downregulated. For cellular components, the upregulated DEGs were mainly related to the mitochondrion, endoplasmic reticulum, and Golgi apparatus, and downregulated DEGs were mainly enriched in the cytosol, nucleus, and cytoplasm. As for molecular functions, the upregulated DEGs were mainly related to integral components of membrane, metal ion binding, and RNA polymerase II transcription regulatory region sequence-specific DNA binding, while structural components of ribosomes and RNA binding were downregulated.

According to Kyoto Encyclopedia of Genes and Genomes (KEGG) pathway analysis, the upregulated DEGs were highly associated with pathways including endocytosis, protein processing in endoplasmic reticulum, oxidative phosphorylation, and ubiquitin-mediated proteolysis (Figure 2C, Table S4). The downregulated DEGs were involved in several pathways, including metabolic pathways, biosynthesis of secondary metabolites, biosynthesis of amino acids, ribosome, carbon metabolism, RNA transport, and purine metabolism (Figure 2D, Table S5). However, only endocytosis pathway was significantly enriched analyzed by upregulated genes, there were 10 pathways significantly enriched by downregulated genes and the biosynthesis of amino acids pathway was most significant. There are 116 genes on biosynthesis of amino acids pathway in *M. oryzae*, 95 genes were downregulated with 9-phenanthrol treatment, suggesting 9-phenanthrol disrupted the expressions of genes belonging to biosynthesis of amino acids pathway.

Because of the decreased pathogenicity of *M. oryzae* treated with 9-phenanthrol, we compared the expressions of identified pathogenicity-related genes in our transcriptome data. We found there were 159 pathogenicity-related genes regulated, 71 downregulated genes, and 88 upregulated genes (Table S6). For downregulated genes, *MHP1* (*MGG_10105*) encoding hydrophobin is associated with conidiation and infectious growth in host cells. Cytochrome P450 monooxygenase (*MGG_07626*) regulates the hypha growth. 4-aminobutyrate aminotransferase (*MGG_01662*) influences fungal virulence. Interestingly, three pigment synthesis-related genes [8], *ALB1* (*MGG_07219*), *RSY1* (*MGG_05059*), and *BUF1* (*MGG_02252*), were upregulated, suggesting 9-phenanthrol could impact the pigment formation. Thus, the different expressions of pathogenicity genes indicated the complicated regulation by 9-phenanthrol.

### 2.3. Quasi-Targeted Metabolomic Analyses Underlying the Compound Changes in M. oryzae with 9-Phenanthrol

**Figure 3:**
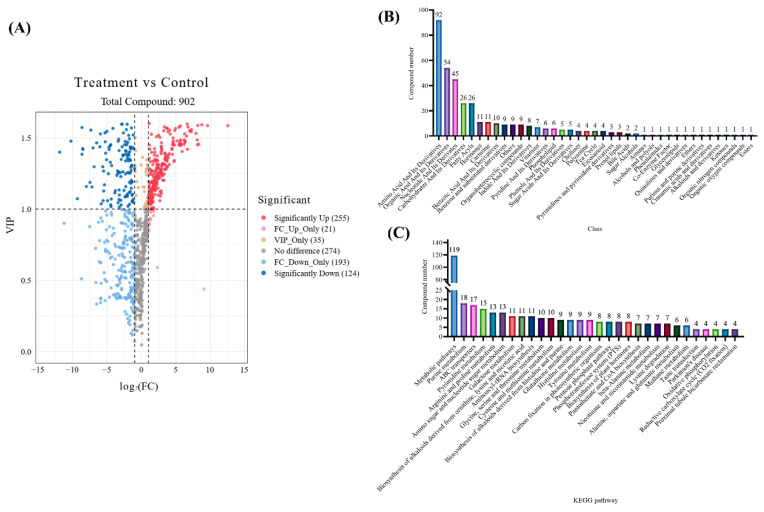
Metabolome analysis reveals the differently changed compounds with 9-phenanthrol in *M. oryzae*. (**A**) The volcano diagram of identified metabolites under 9-phenanthrol treatment. The class (**B**) and KEGG pathways (**C**) of significantly different metabolites of *M. oryzae* under 9-phenanthrol treatment.

We found that metabolic pathway-related genes were significantly changed with 9-phenanthrol treatment through transcriptome analysis. Thus, we used the quasi-targeted metabolome to analyze the variations of compounds in *M. oryzae* with 9-phenanthrol. There were 902 metabolites obtained, and 379 compounds were significantly down- or upregulated (Figure 3A, Table S7). There were 39 classes identified from these significantly different metabolites and most of which were amino acids, organic acids, nucleotides, carbohydrates, and fatty acyls related compounds (Figure 3B). KEGG analysis revealed that the top 10 enriched pathways were metabolic pathways, biosynthesis of secondary metabolites, purine metabolism, ABC transporters, pyrimidine metabolism, arginine and proline metabolism, amino sugar and nucleotide sugar metabolism, galactose metabolism, biosynthesis of alkaloids derived from ornithine, lysine and nicotinic acid, and aminoacyl-tRNA biosynthesis (Figure 3C). Moreover, metabolic pathways, biosynthesis of secondary metabolites, purine metabolism, and pyrimidine metabolism were both enriched in the KEGG terms from transcriptome and metabolome results.

### 2.4. Overexpression of 4-Hydroxyphenylpyruvate Dioxygenase Enhances the Tolerance to 9-Phenanthrol and Other Stresses in M. oryzae

**Figure 4:**
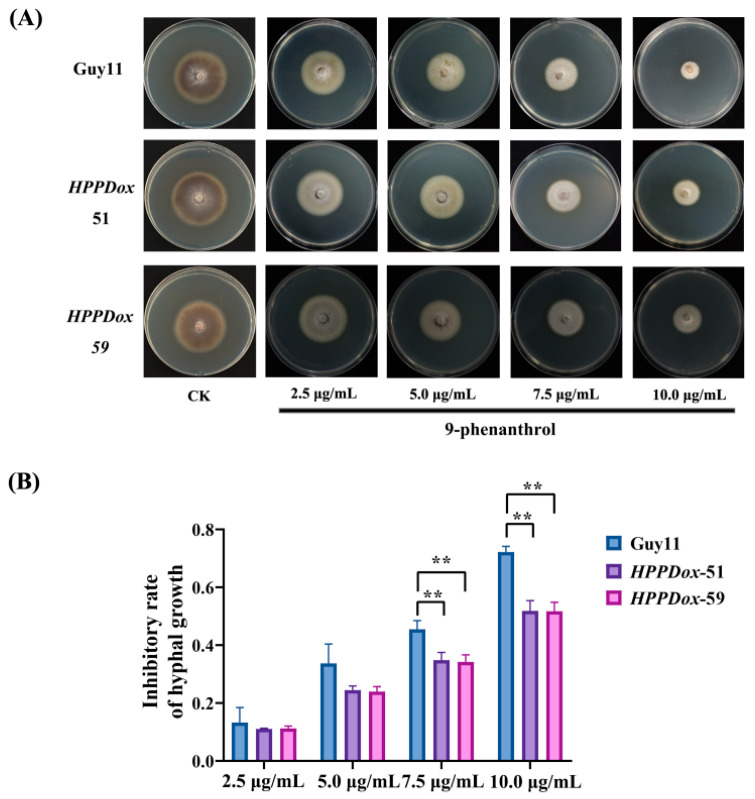
Overexpression of 4-hydroxyphenylpyruvate dioxygenase (HPPD) enhances the tolerance of 9-phenanthrol in *M. oryzae*. (**A**) The colony appearance of HPPD overexpression strains and WT under 9-phenanthrol treatment. (**B**) The inhibitory rates of hyphal growth of *M. oryzae* under 9-phenanthrol treatment. Values are presented as the mean of results of triplicate experiments ± SD. The significant differences between treatments were calculated by Student *t*-test, ** *p* < 0.01.

According to the transcriptomic results, some of the much lower expressions of DEGs were involved in xenobiotic and amino acid metabolism, such as xenobiotic compound monooxygenase (*MGG_05555*), enoyl reductase (*MGG_08363*), 4-hydroxyphenylpyruvate dioxygenase (HPPD) (*MGG_06691*), pescadillo (*MGG_01183*), and ATP-dependent RNA helicase MAK5 (*MGG_00560*). Thus, we cloned these genes with strong promoters and transformed them into Guy11. The transformants were selected under 9-phenanthrol stress. Intriguingly, we found that two transformants overexpressing 4-hydroxyphenylpyruvate dioxygenase showed higher tolerance to 9-phenanthrol (Figure 4A,B).

### 2.5. 9-Phenanthrol Inhibits Germination of Rice and Growth of Other Fungi

**Figure 5:**
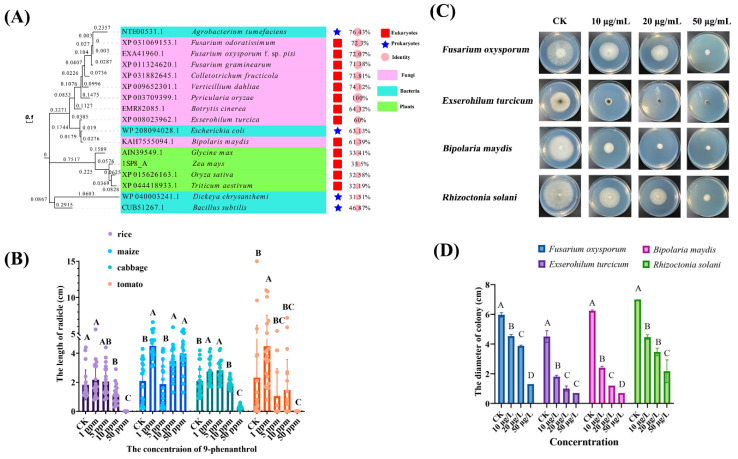
The inhibitory effects of 9-phenanthrol on different plants and fungi. Polygenetic analyses of HDDP in fungi, bacteria, and plants (**A**). Inhibitory rates of 9-phenanthrol on plant germination (**B**) and other fungal pathogens (**C**,**D**). Values are presented as the mean of results of triplicate experiments ± SD. Bars with different letters indicate significant differences between treatments at a *p*-value < 0.01 using a Duncan statistics method.

Due to the wide distribution of HPPD in diverse organisms, we aligned the amino sequences of HPPD in bacteria, plants, and fungi. To our surprise, the species with the most sequence identity of HPPD is *Agrobacterium tumefaciens* compared with that in *M. oryzae*. The polygenetic results showed that most HPPD sequences in fungi were clustered together, and HPPDs in plants belonged to another clade. For bacteria, the HPPDs in *Dickeya chrysanthemi* and *Bacillus subtilis* were clustered, but HPPDs in *A. tumefaciens* and *Escherichia coli* were spread into a fungal clade (Figure 5A). We tested the inhibitory effects of 9-phenanthrol on plants and other fungi, and the results showed that 9-phenanthrol at 10 μg/mL could inhibit the radicle growth of rice and cabbage but had no effect on maize and cabbage (Figure 5B). Moreover, 9-phenanthrol also inhibits the growth of other phytopathogens (Figure 5C,D). While there were no significant inhibitory effects of 9-phenanthrol on *E. coli* and *Pseudomonas aeruginosa*, the *D. chrysanthemi* causing soft rot disease was inhibited at much higher concentrations (Figure S1). *Rhizoctonia solani* and *P. aeruginosa* do not contain putative HPPDs through alignment, other 9-phenanthrol targets and detoxication are existed in *R. solani* and *P. aeruginosa*, respectively.

### 2.6. Gene Co-Expression Network Analysis

**Figure 6:**
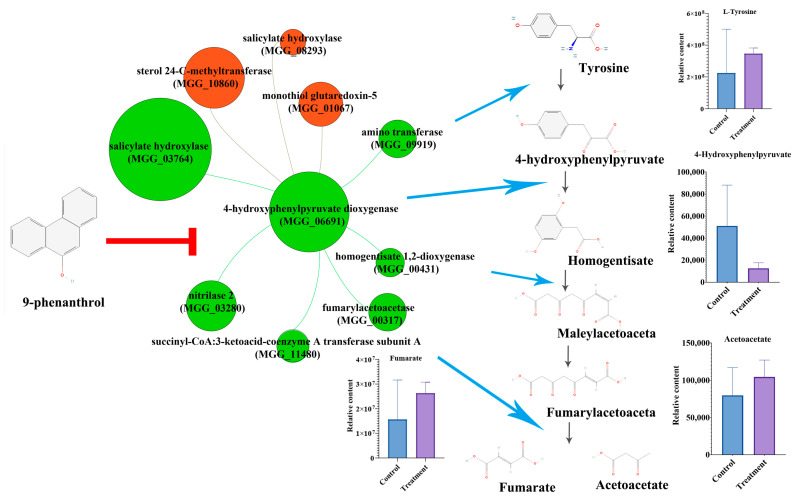
HPPD interacted with gene network and metabolites on the tyrosine degradative pathway. Red nodes represent upregulated genes, and green nodes represent downregulated genes. The size of the node means the absolute value of gene expression. The blue arrows indicate the gene encoding enzyme involving catalyzed reaction among tyrosine degradative pathway. The histograms beside compounds mean the relative contents from metabolome data.

To further analyze the expression pattern of DEGs, we constructed interacted network of DEGs (|foldchange| ≥ 1.5) with 9-phenanthrol treatment using String (Figure S2, Table S9). There are 283 interacted genes, and the most significant interacted genes were RNA and amino acid-associated genes. KEGG analyses of interacted genes were metabolic pathways, biosynthesis of secondary metabolites, tyrosine metabolism, and biosynthesis of antibiotics that were similar to the KEGG results of downregulated DEGs. Moreover, the top 30 interacted genes were all downregulated, indicating the suppressed genes with 9-phenanthrol treatment develop a strong network to disrupt fungal growth. There were nine genes that interacted with HPPD (Figure 6). The expressions of genes encoding sterol 24-C-methyltransferase (*MGG_10860*), salicylate hydroxylase (*MGG_08293*) and monothiol glutaredoxin-5 (*MGG_01067*) were upregulated, succinyl-CoA:3-ketoacid-coenzyme A transferase subunit A (*MGG_11480*), amino transferase (*MGG_09919*), fumarylacetoacetase (*MGG_00317)*, nitrilase 2 (*MGG_03280*), homogentisate 1,2-dioxygenase (*MGG_00431*) and another salicylate hydroxylase (*MGG_03764*) were suppressed. Interestingly, HPPD, amino transferase, fumarylacetoacetase, and homogentisate 1,2-dioxygenase are the key catalyzed enzymes for the tyrosine degradation pathway [9]. We also searched the metabolic products in the metabolome results, and L-tyrosine, 4-hydroxyphenylpyruvate, fumaric acid, and acetoacetate were found. The relative content of 4-hydroxyphenylpyruvate was lower in *M. oryzae* with 9-phenanthrol compared with control. Thus, our results showed that 9-phenanthrol could disrupt the expressions of genes involving the tyrosine degradation pathway in *M. oryzae*.

## 3. Discussion

Rice crop is a staple food around the world. In order to meet its ever-increasing demand, high quantities of chemical pesticides and fertilizers are used. In this paper, we found a novel compound named 9-phenanthrol inhibiting fungal growth and pathogenicity and revealed the fungal toxicity mechanism through transcriptome and metabolome approaches.

### 3.1. 9-Phenanthrol Inhibits Fungal Growth through Transcriptomic Reprogramming

9-phenanthrol is widely used as a specific inhibitor of TRPM4, a Ca^2+^-activated non-selective cation channel, which is associated with cardiac electrical activity, exerts antiarrhythmic effects, and pharmacological effects [5]. As for antifungal activities, there was only one paper that suggested that 9-phenanthrol could change the colony color of *S. sarcinaeforme* and inhibit the spores of *M. fructicola* [7]. We found that 9-phenanthrol had the inhibitory ability to *M. oryzae*, and the inhibitory concentration was equivalent to most antibiotic compounds published [10]. Moreover, we aligned the amino acid sequence of TRPM4 in *M. oryzae*, and no significant similarity was found. Thus, deepening the inhibitory mechanism of 9-phenanthrol against *M. oryzae* is important for transcriptomic analyses (Figure 2A–D).

Endocytosis pathway-related genes were significantly upregulated and enriched according to KEGG analysis, which was involved in the transportation of secretory materials into the cell. SNAREs proteins are composed of major components mediating vesicle fusion, intracellular transport, and plasma membrane fusion involving endocytosis, development, and pathogenesis of *M. oryzae* [11,12]. Antifungal hexapeptide PAF26 shows fungicidal activity against *Neurospora crassa* through the endocytosis pathway, deletion mutants of the endocytic proteins RVS-161, RVS-167 and RAB-5 reduced the rates of PAF26 internalization and fungicidal activity [13]. These results indicate that the entrance of 9-phenanthrol into the cell might regulate the endocytosis pathway.

Downregulated genes were most significantly enriched in the biosynthesis of amino acids pathway. Genes involving arginine, asparagine, cysteine, glutamate, leucine, histidine, methionine, ornithine, serine, threonine, and tyrosine were downregulated with 9-phenanthrol treatment, indicating that 9-phenanthrol inhibits fungal growth by disrupting normal amino acid metabolism (Figure 2D). In *M. oryzae*, there were many genes involving amino acid metabolism identified. Arginine is important for cell signal transduction, protein synthesis, sexual reproduction, biosynthesis genes, *MoCpa1* (*MGG_01743*), *MoARG1* (*MGG_15868*), *MoARG5,6* (*MGG_02690*), and *MoARG7* (*MGG_04210*) are essential for arginine biosynthesis, fungal development, and infection in *M. oryzae*, which were all downregulated with 9-phenanthrol treatment [14,15]. Loss of asparagine synthetase (*MGG_00969*, log_2_FC = −1.38), blast fungus could not grow on the minimal media and virulence defect [16]. Glutamate homeostasis plays a vital role in central nitrogen metabolism. Glutamate synthase *MoGlt1* (*MGG_07187*, log_2_FC = −1.91) mediated glutamate homeostasis is important for autophagy, virulence, and conidiation in the rice blast fungus [17]. Leucine biosynthesis pathway-related genes, *Leu1* (*MGG_01553*), *Leu2A* (*MGG_05223*), *Leu4* (*MGG_13485*) were downregulated in response to 9-phenanthrol [18]. Lysine biosynthesis gene, *MoLys2* (*MGG_02611*, log_2_FC = −1.53) is associated with conidiogenesis and pathogenicity [19]. Moreover, the metabolome results showed that amino acid-related compounds were regulated. Thus, these amino acid-related genes downregulated and changed the contents of compounds by 9-phenanthrol, causing abnormal fungal growth.

### 3.2. 9-Phenanthrol Inhibits Fungal Growth through Disruption of Tyrosine Degradation Pathway

Transcriptome and metabolome analyses showed that the expression of HPPD and the content of 4-hydroxyphenylpyruvate were decreased (Figure 6). Overexpressions of HPPD enhance the tolerance of blast fungus against 9-phenanthrol (Figure 4). HPPD catalyzes the conversion of 4-hydroxyphenylpyruvic acid (HPPA) into homogentisic acid (HGA) in the tyrosine degradation pathway suggesting the relationship between 9-phenanthrol and HPPD. Many commercial herbicides targeting HPPD disrupt carotenoid biosynthesis and cause photosynthetic chlorophyll damage in weeds [20]. However, HPPD is widely conserved in plants, animals, and microbes. In *Aspergillus nidulans*, 4-HPPD deletion mutant could not grow in the presence of phenylalanine and accumulated increased concentrations of tyrosine and 4-hydroxyphenylpyruvic acid [21]. Moreover, we found a higher sequence similarity of HPPD among fungi, which was consistent with higher inhabitation to other fungi (Figure 5). However, the HPPD in *E. coli* shows higher sequence similarity. There is no effect of 9-phenanthrol on the growth of *E. coli* and other bacteria (Figure S4), implying there are extra detoxification mechanisms in procaryotic organisms.

Due to the role of HPPD on homogentisic acid production, HPPD is essential for melanin synthesis [22,23]. Melanin participates in maintaining turgor pressure and facilitates blast fungal infection. Interestingly, we found that three melanin synthesis-related genes, *BUF1*, *ALB1*, and *RSY1* [8], were upregulated in *M. oryzae* with 9-phenanthrol treatment indicating higher expressions of these pigment genes might compensate for the disruption of HPPD by 9-phenanthrol (Table S6). However, there was no evidence that HPPD could directly catalyze 9-phenanthrol or form a complex for detoxification in *M. oryzae*; thus, the relationship between HPPD and 9-phenanthrol needs further research.

Among nine interacted genes, amino transferase, fumarylacetoacetase, and homogentisate 1,2-dioxygenase, together with HPPD, regulate the degradative pathway of tyrosine and were downregulated under 9-phenanthrol treatment (Figure 6). Tyrosine is catalyzed by amino transferase to form 4-hydroxyphenylpyruvate, which can be converted into homogentisate by HPPD. Homogentisate 1,2-dioxygenase catalyzes homogentisate into maleylacetoacetate, and then maleylacetoacetate is converted to fumarylacetoacetate by maleylacetoacetase. Finally, fumarylacetoacetate is cleaved by fumarylacetoacetase to acetoacetate and fumarate [9]. Only maleylacetoacetase encoding gene was not found in our transcriptomic results. The other genes belonging to the tyrosine-degrative pathway were suppressed by 9-phenanthrol treatment. For the other interacted genes, monothiol glutaredoxin-5 is associated with iron homeostasis and iron-sulfur protein maturation [24]. Sterol 24-C-methyltransferase is essential for ergosterol biosynthesis and homeostasis in *Cryptococcus neoformans* [25,26]. Thus, upregulation of these two genes might be an emergency response to induce iron and ergosterol metabolism to cope with 9-phenanthrol stress.

## 4. Conclusions

In this paper, we illuminate the antifungal action mode of 9-phenanthrol on *M. oryzae* by disrupting the expression of HPPD and the content of its metabolite for amino acid dysfunction identified by transcriptome, metabolome, and gene overexpression. Our results provide a novel insight into the toxicity of 9-phenanthrol on *M. oryzae*, which could be referenced by other organisms.

## 5. Materials and Methods

### 5.1. Growth Condition

The rice blast strains stored at −80 °C are activated on the potato sugar agar media at 28 °C in the dark. The solid and liquid complete media supplemented with different compounds were used for the assessment of colony diameter and mycelial dry weight.

### 5.2. Bioactivity Experiments of 9-Phenanthrol

For the antifungal activity experiments, solid CM was added to 2.5 μg/mL, 5 μg/mL, and 10 μg/mL of 9-phenanthrol (CAS: 484-17-3, Sigma-Aldrich, Saint Louis, MO, USA) were used for the inhibitory assessment of *M. oryzae*. The inhibitory rates of 9-phenanthrol on *M. oryzae* were obtained 7 days after inoculation. Other fungal pathogens, such as *F. oxysporum*, *E. turcica*, *B. maydis*, and *R. solani*, were cultivated on potato sugar agar media adding with 2.5 μg/mL, 5 μg/mL, and 10 μg/mL of 9-phenanthrol. The inhibitory rates of 9-phenanthrol were obtained 5 days after inoculation.

For the herbicide activity experiments, the plant seeds were sterilized with 70% (*v*/*v*) ethanol and washed 3 times with sterile water. The seeds were soaked into the sterile water adding 1 μg/mL, 5 μg/mL, 10 μg/mL, and 50 μg/mL of 9-phenanthrol. A total of 4 days after treatment, the lengths of radicle were measured.

### 5.3. RNA Sequencing

The sample preparation for RNA sequencing is referenced by the previous paper [27,28]. The mycelia of Guy11 were cultivated into liquid CM for 2 days and transferred into fresh liquid CM, adding 10 μg/mL 9-phenanthrol for 24 h for RNA extraction. Total RNA was extracted from the tissue using TRIzol^®^ Reagent, and genomic DNA was removed using DNase I (Takara, Tokyo, Japan). The RNA samples were sent to Shanghai Majorbio Technology Co., Ltd. for RNA sequencing based on Illumina HiSeq 6000 platform. The raw data have been deposited on NCBI (Project ID: PRJNA797246).

For read mapping, the clean reads were separately aligned to the reference genome (https://www.ncbi.nlm.nih.gov/assembly/GCF_000002495.2 (accessed on 14 October 2011)) with orientation mode using HISAT2 (http://ccb.jhu.edu/software/hisat2/index.shtml (accessed on 24 July 2020)) software. The mapped reads were assembled by StringTie (https://ccb.jhu.edu/software/stringtie/index.shtml?t=example (accessed on 21 April 2020)).

To identify DEGs (differential expression genes) between two different samples, differential expression analysis was performed using DESeq2 with *p*-adjust < 0.05 and |log_2_FC| ≥ 1. GO and KEGG were performed by DAVID (https://david.ncifcrf.gov/tools.jsp (accessed on 13 November 2020)).

### 5.4. Quasi-Targeted Metabolomic Analyses

Quasi-targeted metabolomics analysis was performed by Novogene Bioinformatics Technology Co., Ltd. (Beijing, China). The sample preparation is consistent with RNA sequencing sample preparation. The harvest mycelium was individually grounded with liquid nitrogen and resuspended with prechilled 80% methanol. The samples were subsequently transferred to a fresh Eppendorf tube and then were centrifuged at 15,000× *g*, 4 °C for 20 min. Finally, the supernatant was injected into the LC-MS/MS system analysis.

LC-MS/MS analyses were performed using an ExionLC™ AD system (SCIEX) coupled with a QTRAP^®^ 6500+ mass spectrometer (SCIEX) in Novogene Co., Ltd. (Beijing, China). The positive transformants were selected and confirmed by PCR and Sanger sequencing. The detection of the experimental samples using MRM (multiple reaction monitoring) was based on a novogene in-house database. The Q3 was used for metabolite quantification. The Q1, Q3, RT (retention time), DP (declustering potential), and CE (collision energy) were used for the metabolite identification. The metabolites with variable importance in the projection, VIP ≥ 1 and |Log2FC| ≥ 1, were considered to be differential metabolites.

### 5.5. Overexpression Strain Construction

The vector pDL2 with the strong constitutive *RP27* promoter was used for overexpressing vector construction. The full length of the candidate gene coding region was amplified from Guy11 genomic DNA and cloned into pDL2 to generate the recombinant vectors using the yeast gap repair approach (Table S9). The correct recombination vector was confirmed by Sanger sequencing and then transformed into *M. oryzae* YN2046 protoplast with PEG-mediated transformation.

### 5.6. Polygenetic Tree Construction

For alignment of HPPD in different organisms, we downloaded the amino acid sequences from NCBI through BLASTP programs, and the polygenetic tree was constructed by MEGA 6.0 using a maximum likelihood method.

## Data Availability

The transcriptome data sets can be retrieved from the NCBI SRA database under Project ID PRJNA797246.

## Supplementary Materials

The following supporting information can be downloaded at: https://www.mdpi.com/article/10.3390/ijms23137116/s1.
