# Supplementary material for: Transcriptome and Quasi-Targeted Metabolome Analyze Overexpression of 4-Hydroxyphenylpyruvate Dioxygenase Alleviates Fungal Toxicity of 9-Phenanthrol in Magnaporthe oryzae
Source: Int J Mol Sci. 2022 Jun 27;23(13):7116. doi: 10.3390/ijms23137116 (PMC9266922; doi:10.3390/ijms23137116)
Supplement: Supplementary file 1 [file ijms-23-07116-s001.zip › Supplementary figures.pdf]

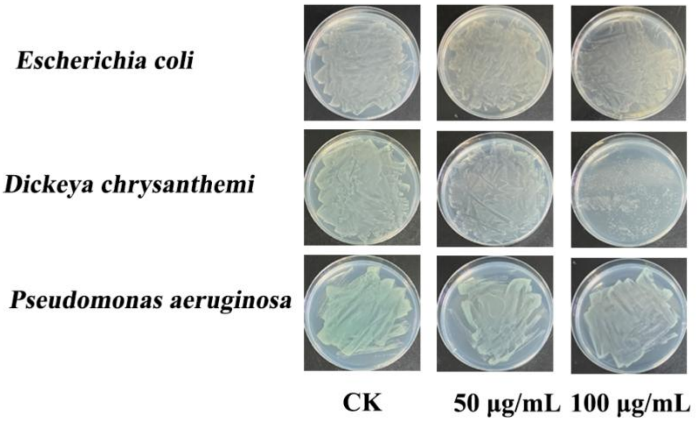

Figure S1. The colony of different bacteria with 9-phenanthrol treatment.

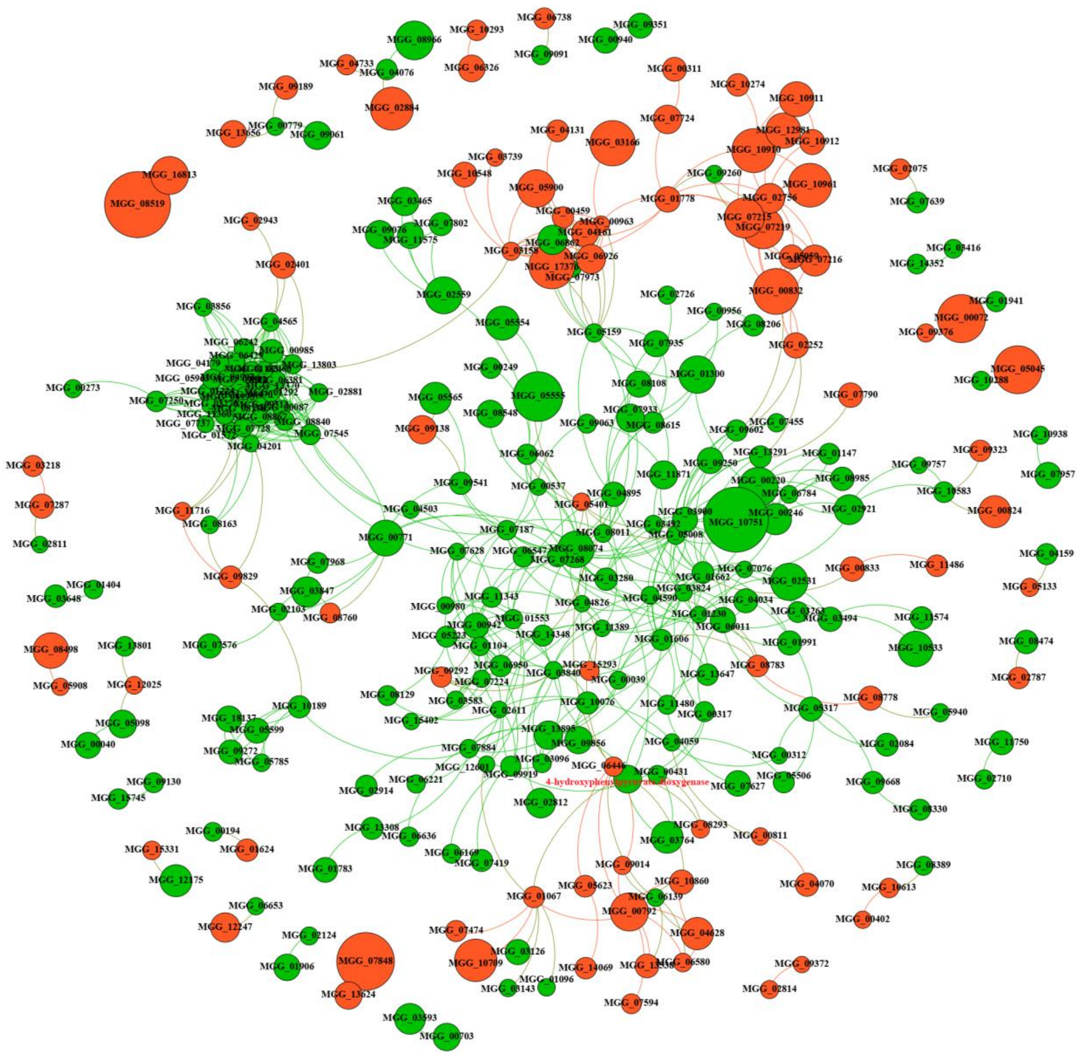

Figure S2. The predicted DEGs interaction network.
